# Relational Well‐Being in Non‐WEIRD Societies: A Reformulation of Happiness From a Chinese Cultural Perspective

**DOI:** 10.1002/pchj.70121

**Published:** 2026-08-31

**Authors:** Mei Xie, Yanhui Mao, Frederick T. L. Leong

**Affiliations:** ^1^ School of Foreign Languages Southwest Petroleum University Chengdu China; ^2^ Institute of Applied Psychology, Psychological Research and Counseling Center Southwest Jiaotong University Chengdu China; ^3^ Division of Applied Psychology, School of Humanities and Social Science Chinese University of Hong Kong Shenzhen China

**Keywords:** Chinese culture, cross‐cultural psychology, happiness, positive psychology, relational well‐being, WEIRD populations

## Abstract

Positive psychology has made substantial contributions to the scientific understanding of human flourishing, yet its conceptual architecture remains disproportionately grounded in WEIRD (Western, educated, industrialized, rich, democratic) populations. Prevailing models privilege intrapersonal processes—autonomy, self‐efficacy, and personal achievement—while systematically under‐theorizing the relational and moral dimensions through which happiness is experienced and evaluated in non‐WEIRD societies. This article proposes a reformulation of happiness through the construct of relational well‐being: a multi‐level framework that reconceptualizes flourishing as emerging from the dynamic interplay between individuals and their embeddedness in social, relational, and moral worlds. Specifically, we define relational well‐being as a pattern of flourishing constituted by four interdependent dimensions: (a) relational connectedness and belonging, (b) role fulfillment and reciprocal obligation, (c) interpersonal harmony and conflict regulation, and (d) moral embeddedness within social and cultural systems. Although these dimensions are not culturally exclusive, we argue that they are normatively foregrounded, elaboratively articulated, and more systematically organized in Chinese cultural contexts—making Chinese society a theoretically generative case for advancing a non‐WEIRD account of well‐being. Drawing primarily on traditional Chinese philosophical traditions—including Confucian, Taoist, and Buddhist perspectives—while acknowledging the dynamic transformation of contemporary Chinese culture, we develop a culturally grounded account of how relational processes constitute—rather than merely contextualize—the experience of happiness. Moving beyond a binary opposition between Chinese and Western models, we adopt a relative emphasis perspective that acknowledges cross‐cultural overlap while mapping systematic divergences in the normative organization of well‐being. We further address key methodological challenges in studying relational well‐being, including construct validity, measurement equivalence, and the risk of conceptual ethnocentrism, and we outline a research agenda for developing culturally sensitive, multi‐level assessment strategies. This article contributes to the ongoing project of building a genuinely inclusive positive psychology—one that integrates individual, relational, and collective dimensions of human flourishing and holds empirical validity beyond the WEIRD populations.

## 1 Introduction

Over the past 25 years, positive psychology has substantially advanced the scientific understanding of human strengths, flourishing, and optimal functioning (Seligman and Csikszentmihalyi 2000). However, its empirical foundation remains disproportionately based on WEIRD (Western, educated, industrialized, rich, democratic) populations (Henrich et al. 2010). Subsequent reviews have confirmed that research on well‐being continues to rely heavily on WEIRD samples, while non‐Western cultural contexts remain comparatively underrepresented (Hendriks et al. 2019; Steger 2025; van Zyl et al. 2024). This imbalance raises an important concern: theories of happiness derived primarily from individualistic societies may not fully capture culturally embedded forms of well‐being in other contexts (Krys, de Almeida, et al. 2025). To develop a more globally relevant positive psychology, greater attention must be given to how culture shapes both the meaning and experience of well‐being (Schimmelpfennig et al. 2024).

Reflecting their cultural origins, dominant models of happiness in positive psychology typically emphasize autonomy, self‐efficacy, personal achievement, and self‐regulation (Diener et al. 2018; Ryan and Deci 2000). Although these models have generated substantial empirical support, they primarily foreground intrapersonal processes and comparatively under‐theorize the relational and moral dimensions of flourishing (Christopher et al. 2014; Fowers 2016; Reis and Gable 2015; Wissing et al. 2021). In contrast, growing cross‐cultural research suggests that well‐being in many non‐WEIRD societies is closely intertwined with interpersonal relationships, social harmony, and role‐based responsibilities (Gardiner et al. 2020; Lu and Gilmour 2004; Uchida and Kitayama 2009). These differences should not be viewed as categorical distinctions between cultures, but as variations in the relative emphasis placed on individual and relational dimensions of well‐being (Krys et al. 2023).

China provides a theoretically informative context for examining these relational dimensions. While modernization and globalization have strengthened individual aspirations, longstanding relational and moral traditions continue to shape how well‐being is understood and experienced (Singh et al. 2023; Steele and Lynch 2013). The Chinese case does not represent all non‐WEIRD societies; instead, it offers a culturally grounded perspective for examining relational and moral aspects of flourishing that may inform broader cross‐cultural comparisons.

Accordingly, this article develops the concept of relational well‐being as a multi‐level framework integrating individual, interpersonal, and collective dimensions of flourishing (Bronfenbrenner 1979). Drawing primarily on Confucian, Taoist, and Buddhist traditions alongside cross‐cultural evidence, we argue that relational well‐being is constituted through four interrelated dimensions: relational connectedness, role fulfillment, interpersonal harmony, and moral embeddedness. We further discuss the conceptual, methodological, and practical implications of this framework for advancing a more culturally inclusive positive psychology.

## 2 Happiness in WEIRD Contexts: The Individualistic Paradigm

### 2.1 The Western Conception of Happiness

The foundational theories of happiness in positive psychology have emerged predominantly from Western intellectual traditions that treat the individual as the primary unit of analysis. The subjective well‐being (SWB) framework (Diener 1984) conceptualizes happiness as life satisfaction, positive affect, and the relative absence of negative affect, emphasizing individuals' subjective evaluations of their own lives. Subsequent developments broadened this perspective. Seligman's PERMA model (2011) incorporates positive emotion, engagement, relationships, meaning, and accomplishment, while self‐determination theory (SDT) (Ryan and Deci 2000) identifies relatedness, alongside autonomy and competence, as a fundamental psychological need.

Despite these advances, relational elements remain largely conceptualized as contributors to individual functioning rather than as constitutive features of well‐being itself. In PERMA, relationships primarily support individual flourishing, whereas in SDT, relatedness is treated as a psychological need whose satisfaction facilitates personal growth and self‐regulation. Consequently, these influential models continue to foreground intrapersonal processes while comparatively under‐specifying the relational, moral, and structurally embedded dimensions of well‐being (Fowers 2016; Reis and Gable 2015; Wissing et al. 2021).

This emphasis reflects broader Western philosophical traditions that associate happiness with autonomy, self‐actualization, and the realization of individual potential (Waterman 1993). Accordingly, well‐being is commonly understood as an individual psychological state achieved through self‐regulation and personal fulfillment, with social relationships functioning primarily as resources for enhancing individual outcomes rather than as contexts in which well‐being is co‐constructed (Sharma et al. 2017).

This individual‐centered orientation is also reflected in positive psychological interventions, such as gratitude journaling, self‐compassion, and mindfulness, which predominantly target individual cognition, emotion, and behavior (Duan et al. 2022; Lyubomirsky et al. 2005). Although these interventions have demonstrated substantial benefits, their effectiveness appears less consistent in contexts where relational harmony, social obligations, and collective considerations play a more central role in well‐being (Ng and Lim 2019; Ng and Ong 2022).

Importantly, this discussion does not suggest that Western theories neglect relationships altogether. Instead, the distinction lies in the relative emphasis placed on relational processes. Existing models acknowledge the importance of relationships but generally conceptualize them as antecedents or resources that support individual well‐being, whereas the present framework conceptualizes them as constitutive dimensions of flourishing. This limitation provides the rationale for developing a complementary perspective in which well‐being is understood as both an individual and a relationally embedded phenomenon.

### 2.2 The Cultural Assumptions Behind Individualism

The individualistic orientation underlying dominant Western conceptions of happiness reflects broader philosophical traditions rooted in humanism and Enlightenment thought, which emphasize autonomy, rational agency, and personal freedom (Markus and Kitayama 1991). Within this framework, happiness is closely associated with authenticity, personal choice, and the realization of individual potential (Taylor 1989).

Cross‐cultural research suggests that alternative conceptions of the self are emphasized in different cultural contexts. In many East Asian societies, the self is more commonly understood as relational, interdependent, and embedded within social networks, where identity is shaped through roles, obligations, and reciprocity (Hwang 2012; Kwan et al. 1997; Markus and Kitayama 1991). Confucian traditions further emphasize moral personhood through the fulfillment of relational responsibilities within the family and wider social order (Yuan et al. 2023). Importantly, these perspectives should not be interpreted as categorical distinctions between Western and non‐Western societies, but as differences in the relative emphasis placed on independent and interdependent models of selfhood (Markus and Kitayama 1991; Oyserman et al. 2002). Moreover, individualism and collectivism are themselves multidimensional orientations: horizontal and vertical forms differ in the extent to which independence or interdependence is combined with equality or hierarchy (Soh and Leong 2002).

These cultural orientations are reflected in emotional ideals, motivational priorities, and moral conceptions of well‐being. Compared with many Western contexts, where happiness is more strongly associated with autonomy, self‐expression, and personal achievement, East Asian contexts tend to place greater emphasis on relational harmony, family cohesion, gratitude, and moral responsibility (Kitayama et al. 2000; Lu and Gilmour 2004; Sedikides et al. 2026; Suh et al. 1998; Yao 2000). Accordingly, well‐being may be evaluated not only by individual satisfaction but also by the quality of social relationships and the fulfillment of relational obligations.

At the same time, these cultural patterns are dynamic rather than static. Globalization and modernization have fostered increasingly hybrid forms of selfhood that integrate both individual and relational orientations (Maricchiolo et al. 2021). Moreover, excessive emphasis on individual achievement may, under some conditions, contribute to self‐focus, social disconnection, and reduced well‐being (Krys, Kostoula, et al. 2025). These developments underscore the importance of moving beyond simple cultural dichotomies toward a more nuanced understanding of how cultural models of the self‐shape well‐being.

### 2.3 The Limitations of the WEIRD Model of Happiness

The predominance of WEIRD paradigms has shaped positive psychology around culturally specific assumptions concerning happiness and flourishing (Wong and Cowden 2022). Although these models have generated important insights, increasing evidence suggests that their applicability across diverse cultural contexts is more limited than often assumed.

#### 2.3.1 Measurement Bias

Widely used instruments such as the satisfaction with life scale (SWLS) and the positive and negative affect schedule (PANAS) were developed within Western traditions that conceptualize happiness primarily as an individual psychological state (Joshanloo 2014). Although psychometrically robust, these measures may not fully capture relational, communal, and moral dimensions of well‐being emphasized in many non‐WEIRD societies (Gould et al. 2025). Likewise, cross‐cultural differences in independent and interdependent conceptions of happiness may distort international comparisons when assessment focuses primarily on life satisfaction (Kiknadze and Fowers 2023; Krys et al. 2023).

#### 2.3.2 Intervention Mismatch

Positive psychological interventions, including gratitude, self‐affirmation, and strengths‐based approaches, generally target individual cognition and self‐improvement. While effective in many Western contexts, their meaning and impact may differ where well‐being is embedded in social relationships and collective practices (Ng and Ong 2022; Shin et al. 2020). For example, gratitude in Chinese culture is often expressed through reciprocal action (baoda, 报答) rather than solely through individual reflection (Hwang 2012), suggesting the need for culturally adapted intervention strategies.

#### 2.3.3 Divergent Moral Foundations

Western models commonly associate happiness with autonomy and personal fulfillment, whereas Chinese traditions emphasize moral cultivation, relational harmony, and social responsibility (Luo 2021; Yao 2000). These differences reflect variations in the relative integration of moral and psychological dimensions of well‐being rather than mutually exclusive cultural models.

#### 2.3.4 Structural and Ecological Limitations

Individual‐centered approaches also pay comparatively less attention to the broader relational and institutional contexts in which well‐being develops. Family systems, social networks, and cultural norms shape well‐being alongside individual cognition and affect (Christopher et al. 2014; Jensen and Sanner 2021). Consequently, understanding well‐being across cultures requires more multi‐level and context‐sensitive frameworks that incorporate both personal and relational processes.

**FIGURE 1:**
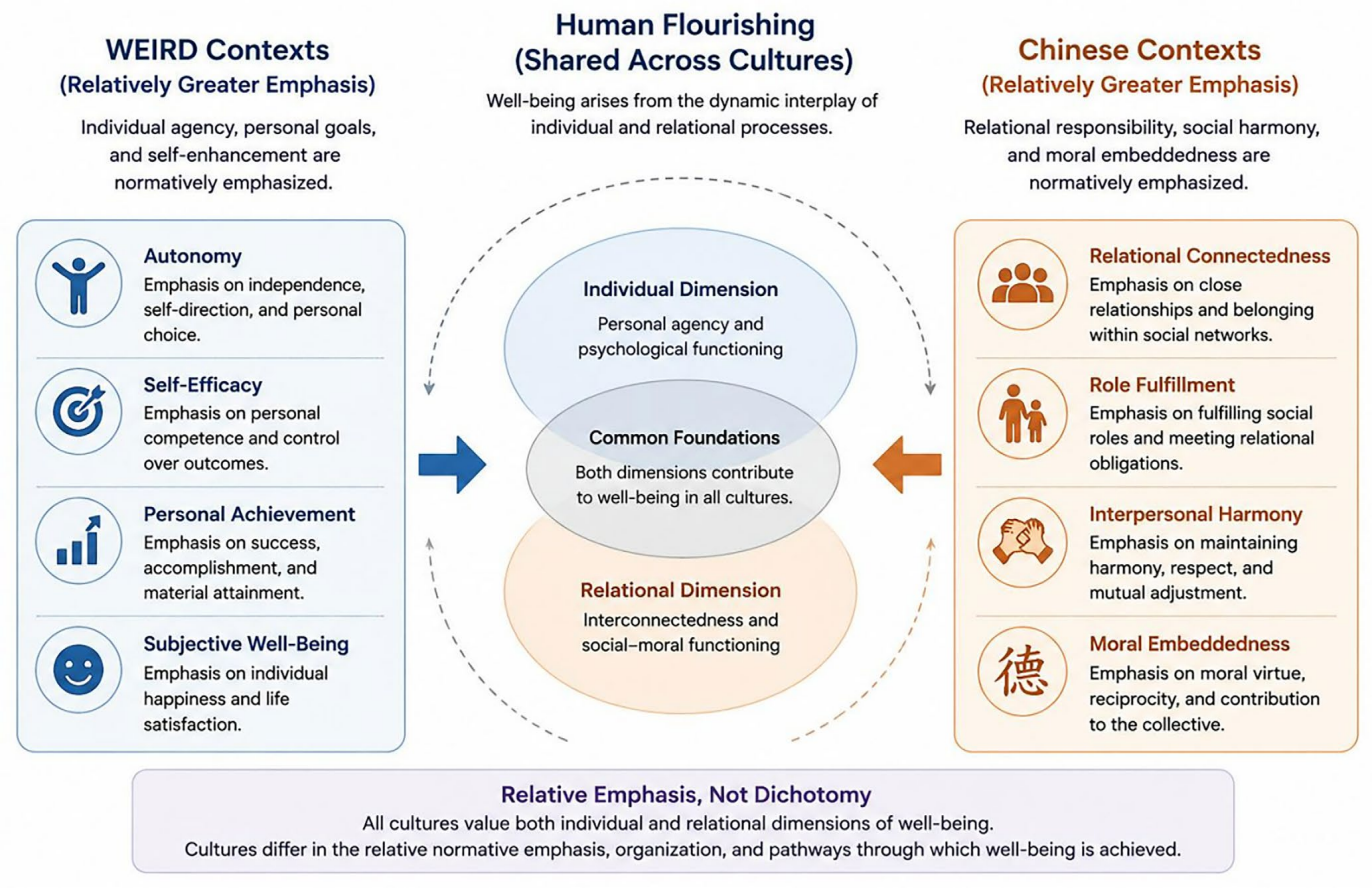
Comparison of WEIRD and non‐WEIRD well‐being paradigms.

Figure 1 illustrates the relative cultural emphases placed on individual and relational dimensions of well‐being. The figure does not depict mutually exclusive paradigms; instead, it highlights that both dimensions are relevant across cultures but differ in their normative prominence and theoretical organization.

## 3 Relational Well‐Being in the Chinese Cultural Context

### 3.1 Defining Relational Well‐Being

Building on the limitations of dominant individual‐centered models, we define relational well‐being as a multi‐level form of human flourishing that emerges through the dynamic interplay between individuals and their social environments (Bronfenbrenner 1979). Specifically, it refers to the co‐construction of meaning, emotion, and value through ongoing social relationships, moral commitments, and culturally embedded practices (Fowers 2016; Reis and Gable 2015).

Relational well‐being comprises four interrelated dimensions: (a) relational connectedness and belonging, reflecting individuals' embeddedness within meaningful social networks; (b) role fulfillment and reciprocal obligation, referring to the enactment of socially and culturally defined responsibilities; (c) interpersonal harmony and conflict regulation, emphasizing empathy, coordination, and relational balance; and (d) moral embeddedness and prosocial orientation, linking well‐being to ethical conduct and contributions to collective welfare. Together, these dimensions capture the principal relational processes through which well‐being is constituted across interpersonal, social, and moral domains. Although analytically distinct, they are empirically interdependent and jointly shape the experience of flourishing.

Importantly, relational well‐being should not be understood as a culturally exclusive construct or as replacing existing theories of well‐being. Instead, it proposes that relational processes constitute a more central and systematically organized dimension of flourishing in some cultural contexts, particularly within Chinese traditions.

To clarify its conceptual position, relational well‐being is distinguished from related constructs such as social well‐being, interdependent happiness, relational harmony, family well‐being, and collective well‐being. While these constructs all emphasize social or relational aspects of human functioning, they differ in theoretical focus and level of analysis. In contrast, relational well‐being is proposed as an integrative multi‐level framework that links interpersonal connectedness, role fulfillment, interpersonal harmony, and moral embeddedness within a unified conception of flourishing. Instead of introducing another relational outcome, it conceptualizes well‐being as emerging from the interaction of individual, interpersonal, and socio‐cultural processes. The conceptual distinctions among these constructs are summarized in Table 1.

**TABLE 1:** Conceptual comparison of relational well‐being and related well‐being constructs.

Construct	Primary focus	Level of analysis	Distinctive feature
Social well‐being	Social integration and functioning	Individual–society	Functioning within society
Interdependent happiness	Happiness through harmonious relationships	Individual	Happiness shaped by relational harmony
Relational harmony	Conflict reduction and harmonious interaction	Interpersonal	Relationship quality
Family well‐being	Family functioning	Family	Family relationships
Collective well‐being	Welfare of communities/groups	Collective	Group outcomes
**Relational well‐being (present study)**	**Relational flourishing through connectedness, role fulfillment, harmony and moral embeddedness**	**Multi‐level (individual–interpersonal–collective)**	**Integrative framework linking relational, moral and cultural processes**

The proposed framework is informed primarily by Confucian conceptions of the relational self. Within this tradition, personhood is understood not as an isolated attribute but as an ethical accomplishment realized through participation in structured social relationships (Hwang 2012). The five cardinal relationships (Wu Lun) define reciprocal obligations that locate individuals within a broader moral order. Accordingly, well‐being arises not solely from individual satisfaction but from fulfilling relational responsibilities in accordance with virtue (de) and propriety (li), through which both personal fulfillment and social harmony are sustained (Snell et al. 2022). The Confucian concept of joy (le) therefore reflects an affective outcome of moral participation in a well‐ordered relational system rather than merely an internal emotional state.

Taken together, relational well‐being provides an integrative framework linking individual experience, interpersonal relationships, and broader moral‐cultural contexts. By specifying its four dimensions while grounding them in both psychological theory and Chinese philosophical traditions, the framework offers a clearer foundation for future empirical research and cross‐cultural comparison.

### 3.2 Confucian Roots: Harmony and Benevolence

Building on the relational conception of well‐being, Confucianism provides an important philosophical foundation for understanding how relational well‐being is cultivated in Chinese contexts. Rather than being presented as a universal model, Confucian ethics illustrate a culturally grounded form of flourishing in which moral, relational, and psychological processes are closely integrated.

Central to Confucian thought is he (harmony), understood not as passive conformity but as an actively maintained balance achieved through empathy, reciprocity, and sensitivity to social roles (Chuang 2005; Yao 2000). Within this framework, well‐being is closely tied to fulfilling relational responsibilities in accordance with de (virtue) and li (propriety). Accordingly, the Confucian concept of le (joy) reflects the affective outcome of aligning personal conduct with relational and moral expectations rather than simply experiencing positive emotion (Luo 2019; Yao 2000).

Confucian ethics therefore emphasize self‐regulation, reciprocity, and role fulfillment as foundations of both individual flourishing and social harmony. Empirical studies support these mechanisms. Family interaction patterns consistent with Confucian values predict greater harmony and subjective well‐being (Chuang 2005), while relationship harmony, dialectical coping, trust, and reciprocity have all been linked to psychological adjustment and social capital in Chinese contexts (Wang et al. 2016; Zhao and Roper 2011). More broadly, relational self‐construals are associated with greater life satisfaction, prosocial behavior, and self‐regulation in East Asian samples (Chu and Vu 2022; Park et al. 2017).

Recent developments in Chinese cultural psychology further extend these ideas through the framework of outside roundness and inside squareness (外圆内方; Du et al. 2025), which describes the ability to maintain interpersonal harmony while preserving moral integrity. Although conceptually related to relational well‐being, this framework primarily describes a psychological strategy for balancing social adaptation with ethical commitment. By contrast, relational well‐being is proposed here as a broader multi‐level framework encompassing relational connectedness, role fulfillment, interpersonal harmony, and moral embeddedness. In this sense, Outside Roundness and Inside Squareness may represent one culturally grounded mechanism through which relational well‐being is achieved in Confucian contexts.

At the same time, Confucian ethics are not without tensions. Strong emphasis on role obligations and moral discipline may create pressures associated with conformity, role strain, or emotional suppression (Ramsey 2016). These tensions suggest that relational well‐being involves continual negotiation between personal needs and social expectations, providing a natural transition to the complementary perspectives offered by Taoism and Buddhism.

### 3.3 Taoist and Buddhist Complements

While Confucianism provides the primary moral foundation for relational well‐being, Taoist (Daojia) and Buddhist (Fojiao) traditions offer complementary psychological pathways that enrich this framework. These traditions do not constitute a single unified system; instead, they emphasize different but overlapping mechanisms of regulation, meaning‐making, and self‐transformation.

*Taoism*: flexibility, acceptance, and effortless action. Taoist philosophy associates well‐being with alignment with the Tao, the dynamic order of the natural world. Central concepts such as ziran (naturalness) and wu wei (effortless or non‐coercive action) emphasize adaptive responsiveness rather than excessive control (Ames and Hall 2003; Slingerland 2007). Psychologically, Taoism promotes cognitive flexibility, acceptance, and harmonious adjustment to changing circumstances. Empirical studies similarly link Taoist principles with balance, emotional regulation, and more stable well‐being trajectories (Chen 2025; Wang et al. 2016; Xu 2023). Together, these findings suggest that Taoism primarily supports the dimensions of interpersonal harmony and adaptive self‐regulation within relational well‐being.

*Buddhism*: compassion, decentering, and interdependence. Buddhist philosophy emphasizes mindfulness, compassion, and dependent origination, viewing the self as an interdependent rather than fixed entity. These principles promote decentering, self‐transcendence, and reduced attachment to rigid self‐concepts. Empirical studies show that Buddhist cultural practices foster resilience, compassion, and prosocial behavior while buffering the effects of stress (Chu and Vu 2022; Shiah et al. 2016; Tan et al. 2023). Within the present framework, Buddhism primarily reinforces the dimensions of moral embeddedness and relational connectedness.

Although these traditions differ in emphasis, they are complementary rather than contradictory. Confucianism foregrounds role fulfillment and moral responsibility, Taoism emphasizes adaptive responsiveness, and Buddhism promotes compassion and self‐transcendence. Rather than representing competing philosophies, they provide distinct psychological resources that individuals may draw upon according to situational demands (Kashdan and Rottenberg 2010).

Recent research suggests that these traditions continue to operate jointly in contemporary Chinese society. Confucian duty, Taoist acceptance, and Buddhist compassion have been associated with resilience, adaptive emotion regulation, and psychological well‐being across diverse contexts (Ding et al. 2022; Lai et al. 2025; Lin et al. 2024). Together, the Confucian–Taoist–Buddhist traditions provide complementary pathways through which relational well‐being is cultivated, linking role fulfillment, adaptive regulation, interpersonal harmony, and moral engagement.

### 3.4 Modern Transformations of Relational Well‐Being

While the Confucian–Taoist–Buddhist traditions provide the philosophical foundation for relational well‐being, they operate within a rapidly changing sociocultural context. Urbanization, globalization, digitalization, and economic transformation have reshaped family structures, social networks, and identity formation in contemporary China. Consequently, relational well‐being should be understood not as a fixed cultural inheritance but as a dynamic construct that is continually reinterpreted across changing social contexts.

Traditional guanxi systems have likewise evolved under conditions of greater geographic mobility, marketization, and digital communication (Yang et al. 2023). These developments have expanded opportunities for self‐expression and individual aspiration while preserving the continuing importance of relational obligation and social harmony (Steele and Lynch 2013; Sun and Ryder 2016). Instead of indicating a simple transition from collectivism to individualism, they reflect increasingly complex and context‐dependent patterns of selfhood and well‐being.

Empirical evidence supports this dynamic perspective. Family relationships, social trust, and perceived stability remain important predictors of life satisfaction in Chinese populations (Diener et al. 2018; Lu 2008), although their influence varies across demographic and socioeconomic contexts (Huang et al. 2024; Yu and Chiu 2016). Contemporary expressions of relational well‐being also extend beyond traditional settings. Digital communities, civic participation, and volunteer activities provide new forms of social connectedness, while practices such as mindfulness are increasingly interpreted through local cultural values emphasizing harmony and balance (Lu 2008). Similarly, greater relational mobility has expanded opportunities for forming new relationships while requiring more flexible patterns of social adaptation (Wei et al. 2025).

Together, these developments point to a hybrid and context‐sensitive model of well‐being. Contemporary Chinese culture should not be equated with traditional collectivism. Research documents increasing individualistic values, self‐expression, and personal choice alongside the continued influence of relational traditions (Cai et al. 2018; Hamamura and Xu 2015; Santos et al. 2017). Concepts such as the bicultural self (Lu 2008) capture this integration, whereby individuals increasingly combine moral interdependence with greater autonomy and self‐development.

Accordingly, relational well‐being should be understood as a culturally significant—but not exclusive—pathway to flourishing in contemporary China. The present framework draws primarily on traditional Chinese philosophical resources while recognizing that these relational orientations are continually reshaped through modernization, globalization, and cultural change. Rather than representing a uniquely Chinese or universally non‐WEIRD model, relational well‐being provides a culturally grounded framework that may inform broader comparative research on human flourishing.

**FIGURE 2:**
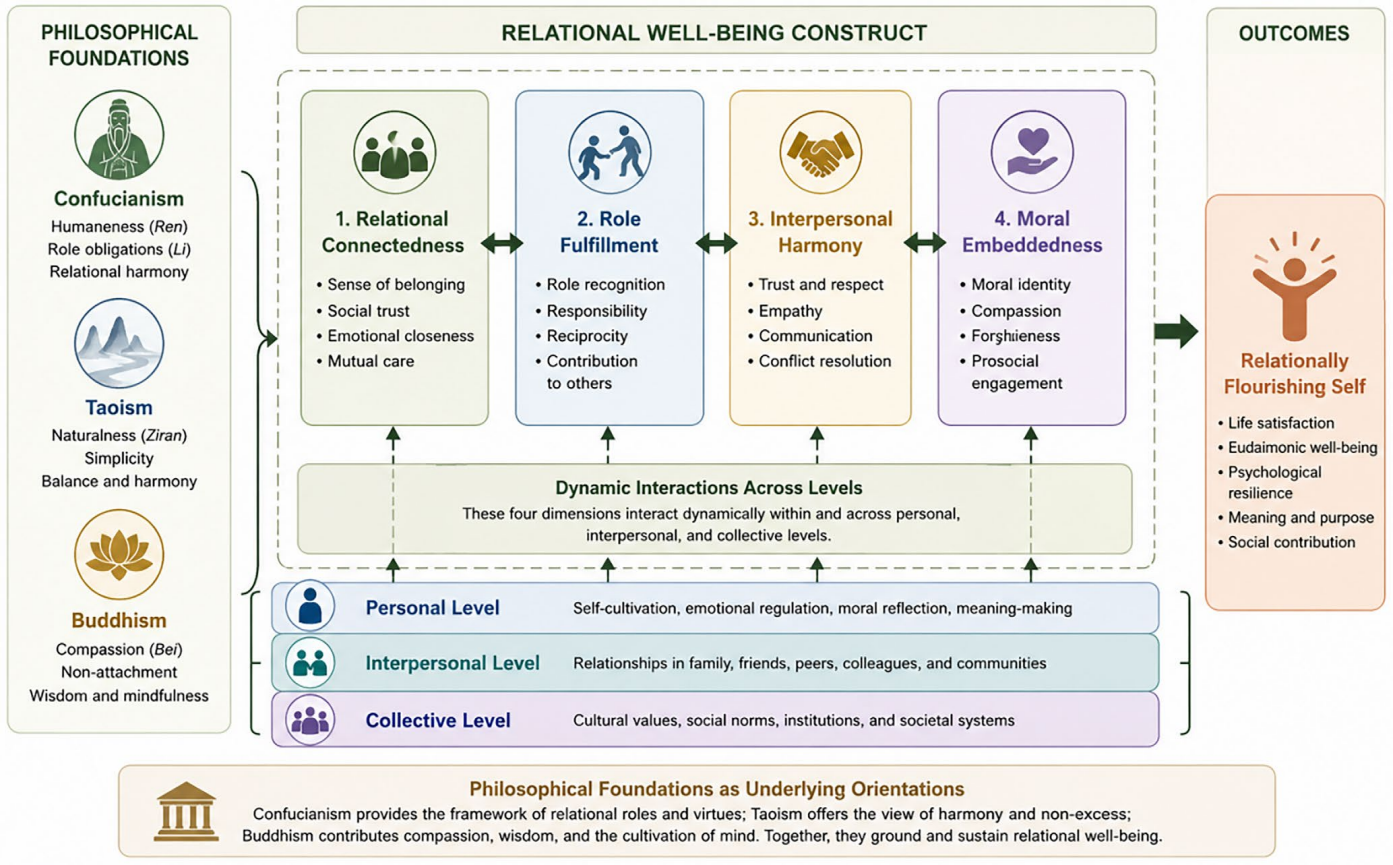
Multi‐level construct of relational well‐being.

Figure 2 illustrates this multi‐level framework, showing how relational connectedness, role fulfillment, interpersonal harmony, and moral embeddedness interact to support flourishing within changing cultural contexts.

## 4 Empirical Insights From Non‐WEIRD Research

### 4.1 Cross‐Cultural Evidence on Relational Happiness

Cross‐cultural research consistently shows that well‐being comprises both universal and culturally embedded components. Instead of supporting culture‐specific models of happiness, this literature suggests that cultures differ primarily in the relative emphasis placed on individual and relational dimensions of flourishing.

Early studies demonstrated that the basis of life satisfaction differs across societies. In more individualistic contexts, happiness is more strongly associated with personal achievement and internal affect, whereas in more collectivist contexts, greater weight is placed on social relationships, role obligations, and interpersonal harmony (Suh et al. 1998). Similarly, Uchida et al. (2004) found that Japanese participants associated happiness with relational harmony and socially engaged emotions, whereas American participants emphasized self‐esteem and personal achievement. Broader cross‐cultural evidence likewise suggests that Western societies tend to prioritize autonomy and pleasure, whereas many Asian, African, and Latin American contexts place relatively greater emphasis on social connectedness and collective well‐being (Delle Fave et al. 2011; Markus and Kitayama 1991).

Importantly, these findings do not imply mutually exclusive models of well‐being. Across cultures, both individual and relational factors contribute to flourishing, although their relative importance varies across social, economic, and cultural contexts (Oishi and Diener 2014). Within the present framework, these findings support the dimensions of relational connectedness, interpersonal harmony, and role fulfillment, while demonstrating that relational well‐being complements rather than replaces individual‐centered models.

Nevertheless, important limitations remain. Cross‐cultural research continues to rely disproportionately on Western‐developed self‐report measures and cross‐sectional designs, raising concerns regarding measurement equivalence and cultural interpretation (Heine et al. 2002). In addition, many non‐WEIRD regions remain underrepresented in comparative research (Krys, de Almeida, et al. 2025). Future studies should therefore employ more culturally sensitive measures, longitudinal and mixed‐method designs, and broader global sampling to better capture the diversity of relational well‐being.

Overall, current evidence supports relational well‐being as an important—but not exclusive—dimension of human flourishing. Rather than reinforcing a dichotomy between individualistic and collectivistic cultures, it highlights culturally varying combinations of individual and relational pathways to well‐being.

### 4.2 Chinese Studies on Relational Well‐Being

Research conducted in Chinese contexts provides growing empirical support for the relational nature of well‐being. Rather than depicting a single model, these studies consistently demonstrate that relational processes contribute to well‐being across family, educational, organizational, and community settings.

Early theoretical work proposed that Chinese well‐being reflects the coexistence of individual‐ and social‐oriented forms of happiness (Lu and Gilmour 2004). Building on this perspective, Wang et al. (2016) identified relationship harmony, non‐attachment, and dialectical coping as key components of indigenous well‐being, highlighting the importance of flexible self‐regulation within relational contexts.

Empirical studies further support the four dimensions of relational well‐being proposed in this article. Relational connectedness is reflected in evidence linking family cohesion, parent–child relationships, and relational needs satisfaction to greater life satisfaction (Chuang 2005; Shek 2000; Yu et al. 2024). Role fulfillment and interpersonal harmony are supported by findings showing that social support, belonging, interpersonal trust, and supportive educational and organizational environments promote well‐being and buffer occupational stress (Tong et al. 2019; Wu and Zhou 2023). At the same time, studies on relational mobility suggest that changing social structures influence well‐being by reshaping opportunities for forming and maintaining social ties (Zhang and Zhao 2021).

Research has also highlighted the importance of moral embeddedness. Relational self‐esteem is positively associated with life satisfaction in collectivist contexts (Du et al. 2017), whereas materialistic values appear to weaken relational aspects of well‐being (Yoo et al. 2021). Together, these findings suggest that ethical values and relational motivations remain important components of flourishing in Chinese society.

Overall, existing evidence supports relational well‐being as a meaningful but context‐dependent framework. Rather than operating independently of individual factors, relational processes interact with personal characteristics, social institutions, and cultural values in shaping well‐being. At the same time, more longitudinal, multi‐method, and cross‐cultural research is needed to clarify the mechanisms and boundary conditions of relational well‐being.

### 4.3 Expanding Positive Psychology Through Relational Constructs

Recent developments in positive psychology have increasingly recognized that flourishing is shaped not only by individual characteristics but also by relational and social processes (Wong 2011). Instead of replacing traditional theories, these developments have expanded positive psychology toward a more context‐sensitive understanding of well‐being.

Relational perspectives have emerged across multiple domains. Research on eudaimonic well‐being highlights the importance of meaningful relationships and prosocial engagement (Ryff and Singer 2000), while studies of social flow, collective mindfulness, and communal resilience demonstrate that cooperation, shared meaning, and social support contribute to both well‐being and adaptive functioning (Ungar 2013; Walker 2010; Weick and Sutcliffe 2015; Wissing 2014a, 2014b). Similarly, relational psychological capital emphasizes interpersonal resources such as trust and compassion in promoting well‐being and performance (Luthans et al. 2013).

Despite these advances, existing relational constructs remain largely context‐specific and fragmented across different levels of analysis. Relationships are typically conceptualized as antecedents or resources for individual well‐being rather than as organizing principles of flourishing. Consequently, the relational, moral, and collective dimensions of well‐being have rarely been integrated within a single conceptual framework.

The concept of relational well‐being proposed here builds on these developments while extending them in two important respects. First, it integrates relational connectedness, role fulfillment, interpersonal harmony, and moral embeddedness into a unified multi‐level framework. Second, it situates these relational processes within broader cultural models of selfhood, drawing primarily on Chinese philosophical traditions while remaining applicable to comparative cross‐cultural research. In this sense, relational well‐being is proposed not as a culturally exclusive alternative to existing theories, but as a more comprehensive framework for understanding how flourishing emerges through the interaction of individual, interpersonal, and socio‐cultural processes.

## 5 Challenges and Future Directions on Happiness

### 5.1 Methodological Limitations

Despite growing recognition of relational and cultural dimensions of well‐being, several methodological challenges continue to constrain research in this area. A primary concern is measurement equivalence. Widely used instruments such as the SWLS and the PANAS were developed within Western frameworks that emphasize individual cognition and affect (Joshanloo 2014; Lambert et al. 2020). Although psychometrically robust, their cross‐cultural applicability requires careful evaluation, particularly regarding whether they adequately capture relational, moral, and collective dimensions of well‐being. Establishing measurement invariance is therefore essential to distinguish genuine cultural differences from variations in interpretation or response styles (Chen 2025; Heine et al. 2002).

A second limitation concerns research design. Much of the existing literature relies on cross‐sectional, individual‐level surveys, limiting understanding of the dynamic and socially embedded nature of relational well‐being. Future research should incorporate longitudinal, dyadic, and multi‐level approaches to examine how relational processes unfold across time and social contexts.

Finally, greater use of mixed‐method and combined etic–emic approaches would strengthen future research. Cross‐cultural research requires attention not only to statistical measurement equivalence but also to conceptual equivalence and the integration of indigenous and comparative perspectives (Leong et al. 2010). Combining standardized measures with qualitative methods can better capture culturally situated meanings of well‐being while facilitating comparisons between universal and culture‐specific processes.

### 5.2 Theoretical Integration

A central theoretical challenge in positive psychology is integrating individual‐centered and relational perspectives on well‐being. Existing models typically emphasize either intrapersonal processes (e.g., affect, cognition, and motivation) or contextual influences (e.g., relationships and culture), while offering limited explanation of how these processes interact across levels. We therefore propose relational well‐being as a multi‐level framework linking personal, interpersonal, and collective processes.

At the personal level, relational well‐being involves emotional regulation, moral evaluation, and self‐cultivation. At the interpersonal level, it is shaped through reciprocity, trust, empathy, and co‐regulation. At the collective level, it is embedded within broader cultural values, social norms, and institutional contexts. These levels are dynamically interconnected, such that interpersonal relationships influence individual well‐being while broader sociocultural contexts shape relational experiences.

This framework complements rather than replaces existing theories such as self‐determination theory and PERMA. Whereas these models primarily treat relationships as contributors to individual flourishing, relational well‐being conceptualizes relational processes as organizing principles through which well‐being is co‐constructed across individual, interpersonal, and cultural contexts. In doing so, it provides a conceptual bridge between positive psychology and culturally grounded perspectives on human flourishing.

Overall, this integrative framework offers a clearer foundation for future empirical research by specifying how relational connectedness, role fulfillment, interpersonal harmony, and moral embeddedness interact across multiple levels to shape well‐being in diverse cultural contexts.

### 5.3 Cultural Modernity and Globalization

Cultural modernity and globalization present new challenges for understanding relational well‐being. Contemporary societies do not simply shift from collectivism to individualism; instead, they increasingly exhibit hybrid patterns of selfhood in which relational obligations and individual aspirations coexist and are emphasized differently across contexts (Steele and Lynch 2013; Sun and Ryder 2016). These developments suggest that well‐being is negotiated across multiple value systems rather than determined by a single cultural orientation.

Future research should examine how relational and individual priorities vary across situations, life stages, and levels of exposure to globalization. Within‐culture comparisons may be particularly valuable for identifying how hybrid identities shape the relative importance of relational and individual dimensions of well‐being.

Digital technologies further reshape relational life by creating new forms of social connection while also introducing challenges such as social comparison and changing patterns of relational commitment (Elmer et al. 2026). At the same time, comparative cross‐cultural research is needed to examine how relational well‐being operates across diverse cultural settings and how cultural, institutional, and technological factors jointly influence its development.

Overall, cultural modernity highlights the need for a dynamic and context‐sensitive understanding of relational well‐being. Rather than viewing traditional and modern values as competing alternatives, future research should examine how they interact to shape flourishing in an increasingly interconnected world.

### 5.4 Toward a Global Positive Psychology Perspective

Advancing positive psychology toward a more globally relevant science of well‐being requires moving beyond single‐culture models toward more inclusive and culturally responsive frameworks. Rather than assuming the universality of existing theories, future research should examine how well‐being is conceptualized, experienced, and measured across diverse cultural contexts while incorporating culturally grounded constructs into theory development (Christopher et al. 2014).

Achieving this goal will require greater international collaboration, broader representation of non‐WEIRD populations, and more culturally sensitive methodologies. Multi‐country research, culturally inclusive measurement, and mixed‐method approaches can strengthen cross‐cultural comparisons while distinguishing universal from culture‐specific aspects of flourishing.

Within this agenda, relational well‐being provides a framework for integrating individual, interpersonal, and collective dimensions of flourishing. Instead of replacing existing theories, it complements them by explicitly incorporating relational connectedness, role fulfillment, interpersonal harmony, and moral embeddedness into models of well‐being. The framework is proposed not as a uniquely Chinese or universally non‐WEIRD model, but as a culturally grounded perspective that can inform comparative research across diverse societies.

**FIGURE 3:**
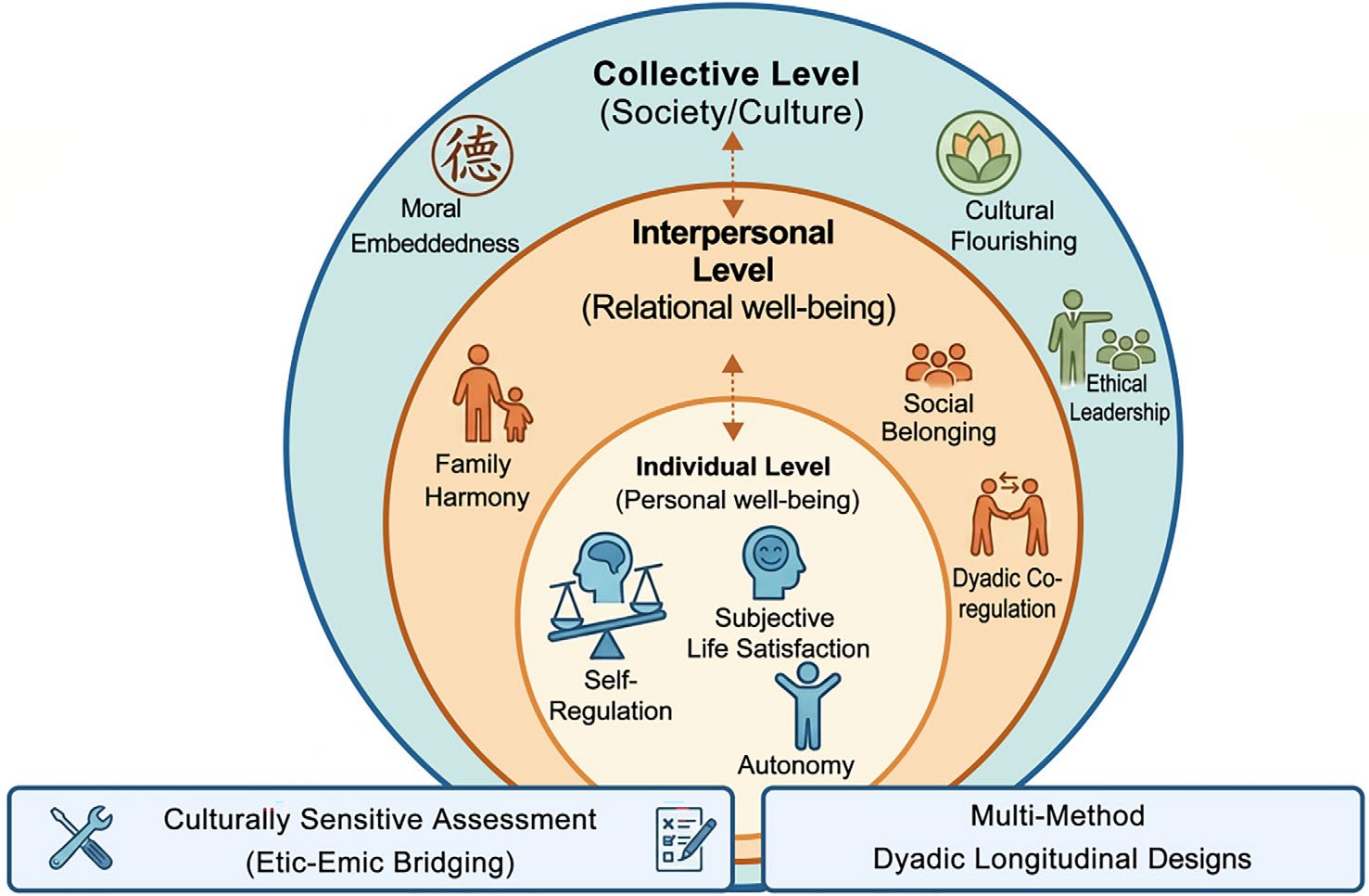
A multi‐level integrative research pathway for global positive psychology.

Ultimately, a global positive psychology should recognize multiple pathways to flourishing while maintaining conceptual clarity and empirical rigor. Figure 3 summarizes this perspective by illustrating how relational well‐being can bridge individual, interpersonal, and collective processes within a more culturally inclusive science of well‐being.

## 6 Conclusion

Positive psychology has greatly advanced the scientific understanding of human flourishing. However, much of its theoretical and empirical foundation has been developed within WEIRD contexts, where well‐being is primarily conceptualized in terms of autonomy, self‐regulation, and individual achievement. While these perspectives remain valuable, they do not fully capture the relational and moral dimensions of well‐being emphasized in many cultural contexts.

This article proposes relational well‐being as a complementary, multi‐level framework integrating relational connectedness, role fulfillment, interpersonal harmony, and moral embeddedness. Drawing primarily on Confucian, Taoist, and Buddhist traditions, we argue that well‐being is co‐constructed through ongoing social relationships and embedded within broader cultural and moral systems. Importantly, this framework is not intended as a uniquely Chinese or universally non‐WEIRD model, but as a culturally grounded perspective that may inform comparative research across diverse societies.

Cross‐cultural and indigenous evidence suggests that relational processes are important contributors to well‐being, although their relative importance varies across cultures and social contexts. Accordingly, relational well‐being should be understood as an important—but not exclusive—dimension of human flourishing that complements existing individual‐centered theories.

Overall, this article contributes to positive psychology by providing a more integrative account of well‐being that connects individual, interpersonal, and collective processes within a single conceptual framework. Future research should continue to evaluate this framework across diverse cultural settings using culturally sensitive and methodologically rigorous approaches. By recognizing both universal and culturally embedded pathways to flourishing, relational well‐being offers a foundation for a more globally inclusive science of well‐being.

## Funding

This work was supported by the National Natural Science Foundation of China (72271205, 71801180) and The Fundamental Research Funds for the Central Universities (2682026GH001, 2682026WGRH009).

## Disclosure

The authors confirm that the AI‐assisted tool (ChatGPT) was used exclusively for linguistic refinement, including grammar correction and stylistic improvement. The intellectual content, research methodology, data analysis, and interpretation were developed entirely by the authors. The authors take full responsibility for the accuracy, integrity, and originality of the work.

## Conflicts of Interest

The authors declare no conflicts of interest.

## Data Availability

Data sharing not applicable to this article as no datasets were generated or analysed during the current study.
